# Dynamic regulation of the cholinergic system in the spinal central nervous system

**DOI:** 10.1038/s41598-020-72524-3

**Published:** 2020-09-18

**Authors:** Mohamad Rima, Yara Lattouf, Maroun Abi Younes, Erika Bullier, Pascal Legendre, Jean-Marie Mangin, Elim Hong

**Affiliations:** 1 INSERM, CNRS, Neurosciences Paris Seine - Institut de Biologie Paris Seine (NPS - IBPS), Sorbonne Université, 75005 Paris, France; 2 Present Address: Institut de Génétique et de Biologie Moléculaire et Cellulaire (IGBMC), INSERM, CNRS, Université de Strasbourg, 67400 Illkirch, France

**Keywords:** Developmental biology, Neuroscience

## Abstract

While the role of cholinergic neurotransmission from motoneurons is well established during neuromuscular development, whether it regulates central nervous system development in the spinal cord is unclear. Zebrafish presents a powerful model to investigate how the cholinergic system is set up and evolves during neural circuit formation. In this study, we carried out a detailed spatiotemporal analysis of the cholinergic system in embryonic and larval zebrafish. In 1-day-old embryos, we show that spinal motoneurons express presynaptic cholinergic genes including *choline acetyltransferase* (*chata*)*, vesicular acetylcholine transporters* (*vachta*, *vachtb*)*, high-affinity choline transporter* (*hacta*) and acetylcholinesterase (*ache*), while nicotinic acetylcholine receptor (nAChR) subunits are mainly expressed in interneurons. However, in 3-day-old embryos, we found an unexpected decrease in presynaptic cholinergic transcript expression in a rostral to caudal gradient in the spinal cord, which continued during development. On the contrary, nAChR subunits remained highly expressed throughout the spinal cord. We found that protein and enzymatic activities of presynaptic cholinergic genes were also reduced in the rostral spinal cord. Our work demonstrating that cholinergic genes are initially expressed in the embryonic spinal cord, which is dynamically downregulated during development suggests that cholinergic signaling may play a pivotal role during the formation of intra-spinal locomotor circuit.

## Introduction

Acetylcholine (ACh) is an ancient molecule found throughout most life forms including bacteria, fungi, plants and animals^1^. In vertebrates, cholinergic neurons, which release ACh during neurotransmission, are found in both the spinal cord and also in distinct areas of the brain, including the basal forebrain, brainstem and the habenula^2^. Brain cholinergic systems regulate many cognitive processes including learning, memory, attention and sleep and as such, has been targeted to treat various diseases including Alzheimer’s, Huntington’s and Parkinson’s diseases^2–5^. During development, cholinergic signaling, the binding of ACh to its receptors, is essential for physiological processes necessary for the formation of the peripheral and central nervous systems (CNS), including spontaneous neuronal activity, axon pathfinding and synaptogenesis^6^. As nicotine binds with high affinity to the endogenous nicotinic ACh receptors (nAChRs) and is able to cross the placenta and the blood brain barrier, many studies have described the detrimental impact of early nicotine exposure on fetal development in humans and animal models resulting in neurobehavioral and locomotion defects^7,8^.

**Figure 1:**
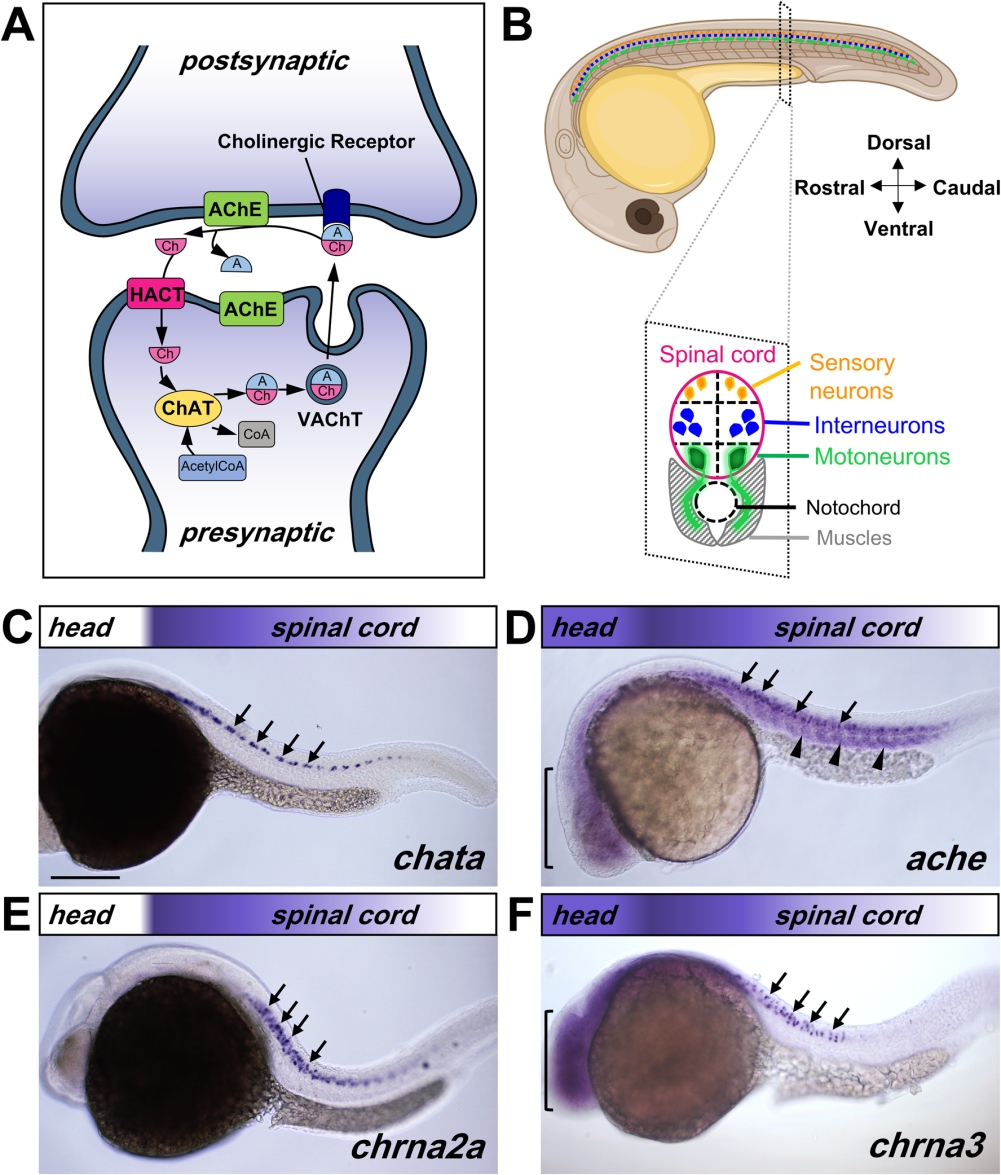
Transcript map of cholinergic genes in the 22–24 hpf embryo. (**A**) Simplified illustration of ACh signaling between pre- and postsynaptic neurons. Choline Acetyltransferase (ChAT), Vesicular Acetylcholine Transporter (VAChT), Acetylcholinesterase (AChE), High Affinity Choline Transporter (HACT), acetyl (A), Choline (Ch), Acetyl Coenzyme A (AcetylCoA). (**B**) Schematic representation of embryonic zebrafish spinal cord organization, showing lateral view of a 24 hpf zebrafish embryo (top), and an illustration of transverse section of the spinal cord (bottom). (**C**–**F**) Lateral view of 22–24 hpf embryos processed by in situ hybridization showing expression for *chata* (**C**) in the spinal cord (arrows), *ache* (**D**) in the spinal cord (arrows), muscles (arrowheads) and the brain (bracket), *chrna2a* (**E**) in the spinal cord (arrows), *chrna3* (**F**) in the spinal cord (arrows) and brain (bracket). The rostro-caudal expression pattern of the transcript is represented by the colored gradient bar on top of each image. Magnification is the same for all images in (**C**–**F**). Scale bar: 200 μm. Cartoon from (**B**) was modified from Biorender.

In the nervous system, cholinergic signaling generally occurs by the coordinated action of the ACh-releasing pre-synaptic and ACh-receiving postsynaptic cells (Fig. 1A). Cholinergic neurons are characterized by the presence of Choline AcetylTransferase (ChAT), which mediates ACh synthesis and the Vesicular AcetylCholine Transporter (VAChT), which loads ACh into vesicles. Presynaptic vesicular release of ACh into the synaptic cleft binds to and activates ACh receptors before being degraded by Acetylcholinesterase (AChE) into acetate and choline. The latter is carried into the presynaptic neurons by the High-Affinity Choline Transporter (HACT). In vertebrates, the effects of ACh is mediated by two pharmacologically-distinct families of receptors; namely, nicotinic and muscarinic ACh receptors (nAChR and mAChR, respectively). Cholinergic signaling has also been shown to occur prior to synapse formation. For example, ACh-release was detected at the growth cone of growing spinal neurons^9^, and ACh somatic exocytosis has also been reported^10^. These findings suggest a functional role of ACh even before the onset of synaptogenesis. As such, it has been shown that cholinergic signaling mediates the emergence of spontaneous neuronal activity during embryonic development of many animal models, and regulates circuit formation in the spinal cord^11–13^. However, how the cholinergic system develops during the early phases of neuronal circuit formation is still poorly understood.

Previous studies have shown that both pre- and postsynaptic cholinergic genes are expressed in the spinal CNS during development. Presynaptic cholinergic genes start being expressed at early embryonic stages in chick (stage 26) and mouse (E10.5)^14,15^ spinal cord. In parallel, nAChRs are also expressed in different regions of the embryonic spinal cord in human, mouse and chick^16–18^. However, a systemic study to investigate whether there are any changes in the cholinergic system throughout spinal CNS development has not been carried out.

The embryonic zebrafish has emerged as an attractive vertebrate model system to study the development and function of neural circuits^19–22^. It is easy to observe and manipulate the embryos during development as they are externally fertilized. In addition, the small size and transparency of the embryo allows the observation of various experimental techniques without the need to dissect the embryo. Therefore, the embryonic zebrafish presents an ideal model system to probe the development of the cholinergic system in the whole organism during early development. Furthermore, the spinal neuronal circuit is well characterized and specific neuronal populations express transcription factors that are conserved in vertebrates^23,24^. In the embryonic zebrafish, motoneurons start expressing *chat* transcript from 16 hours post fertilization (hpf)^25^ and ChAT protein is found in 2-day-old embryos^26^. In addition, *ache* and various nAChR subunit transcripts are present in the spinal cord during early development^27–29^. However, the precise spatial and temporal resolution of many cholinergic genes are still unknown. Therefore, we carried out a detailed analysis on the spatiotemporal expression of the pre- and postsynaptic cholinergic system during early development.

We found high levels of presynaptic cholinergic gene expression in motoneurons and nAChR subunits in interneurons in the embryonic spinal cord, suggesting cholinergic transmission between motoneurons and interneurons in the CNS. During development into larval stages, the cholinergic phenotype in spinal motoneurons is downregulated and gradually becomes restricted to the tail tip, while interneurons continuously express nAChRs throughout the spinal cord. In addition, we found that ChAT-expressing neurons, VAChT puncta on muscles and AChE activity were all decreased in the rostral compared with the caudal spinal cord. Our findings show an unexpected transition of the cholinergic system during development and suggests a pivotal role for cholinergic signaling in the formation of the intra-spinal locomotor circuit.

## Results

### Pre- and postsynaptic cholinergic genes are expressed in the embryonic spinal cord

To understand the spatiotemporal relationship of the different cholinergic genes during early development, we firstly carried out whole mount *in-situ* hybridization (ISH) in 22–24 h post fertilization (hpf) embryos. We found that presynaptic cholinergic genes *chata*, *hacta*, *vachta* and *vachtb* are expressed in the spinal cord of 22–24 hpf embryos (Fig. 1A, C, Supplementary Fig. 1A–C). The presynaptic cholinergic genes *chatb* (Supplementary Fig. 1E) and *hactb* (data not shown) are not expressed in the spinal cord at this stage. nAChR α3 subunit (*chrna3*), α7 subunit (*chrna7*) and α2 subunit (*chrna2a*) transcripts are also expressed in the 22–24 hpf spinal cord (Fig. 1E, F, Supplementary Fig. 1D). *ache* was present in the spinal cord, and ubiquitously expressed in the muscles (Fig. 1D). In the spinal cord, all cholinergic genes show a rostral (high) to caudal (low) expression pattern that coincides with the rostral to caudal sequential development of zebrafish spinal cord^30^.

**Figure 2:**
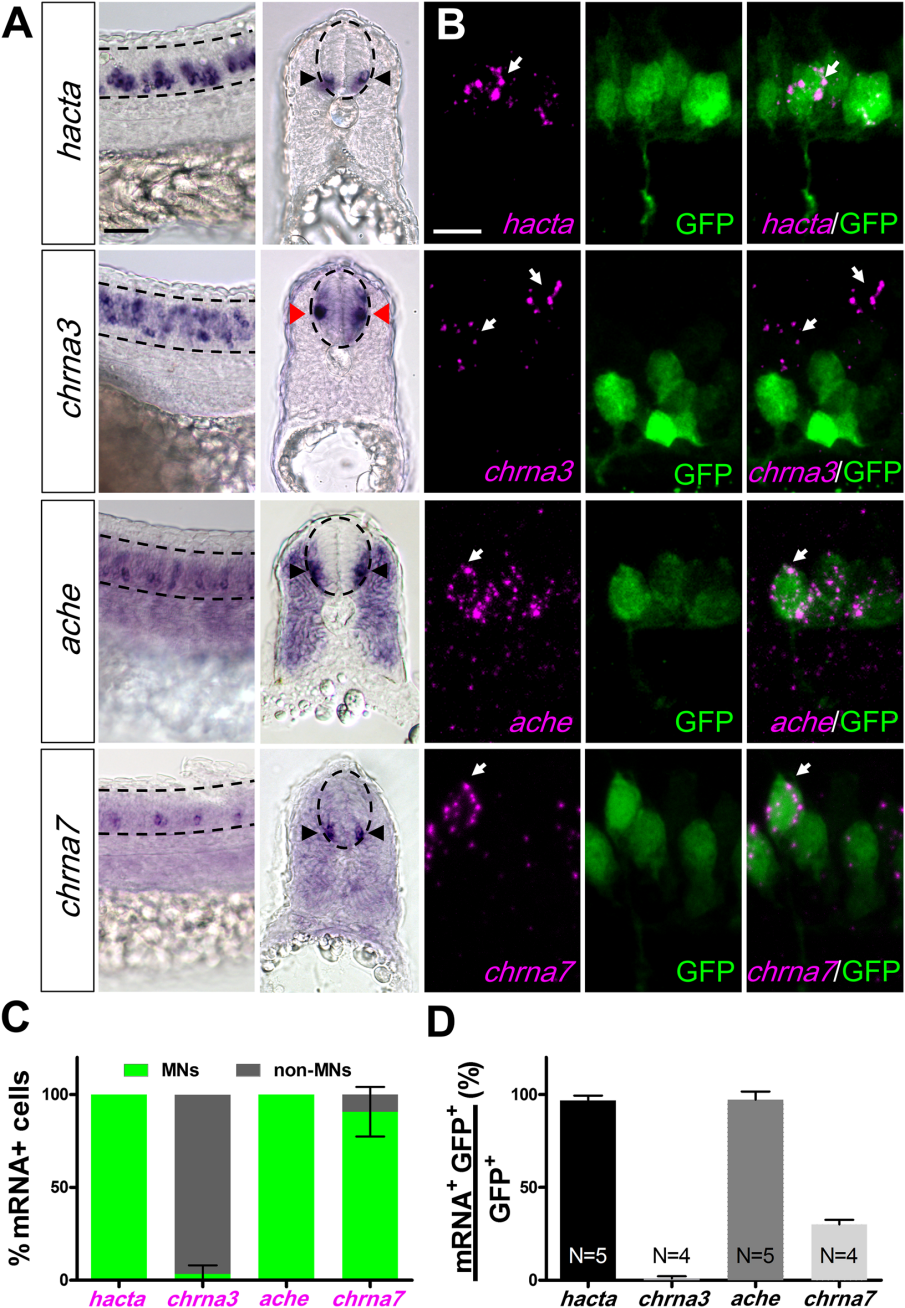
Spatial expression pattern of cholinergic genes in spinal inter- and motoneurons at 24 hpf. (**A**) Lateral view (left) and cross sections (right) of embryos processed by in situ hybridization showing *hacta*, *chrna3*, *ache*, and *chrna7* transcripts in the spinal cord. While *hacta*, *ache*, and *chrna7* transcripts are expressed in the ventral spinal cord (black arrowheads) that contains motoneurons (MNs), *chrna3* is expressed in intermediate spinal cord (red arrowheads) consistent with location of interneurons (INs). The spinal cord is outlined by dashed lines. Scale bar: 30 µm. (**B**) Representative confocal images of fluorescent in situ hybridization of 24 hpf *Tg*(*mnx1:GFP*) embryos showing MNs in green (GFP) and *hacta* (first row), *chrna3* (second row), *ache* (third row), and *chrna7* (fourth row) transcript in magenta (white arrows). Scale bar: 15 µm. (**C**) Classification of *hacta*, *chrna3*, *ache*, and *chrna7* expression in MNs (green) or non-MNs (grey). (**D**) Percentage of *hacta*, *chrna3*, *ache*, and *chrna7* expressing *mnx1:*GFP neurons. *hacta:* N = 5 embryos (176 *mnx1:*GFP^+^ neurons, 171 *hacta*^+^ neurons). *chrna3:* N = 4 embryos (214 *mnx1:*GFP^+^ neurons, 77 *chrna3*^+^ neurons). *ache*: N = 5 (132 *mnx1:*GFP^+^ neurons, 128 *ache*^+^ neurons). *chrna7:* N = 8 embryos (223 *mnx1:*GFP^+^ neurons, 59 *chrna7*^+^ neurons).

In the zebrafish embryo, the spinal cord shows a ventral to dorsal organization consisting of motoneurons, interneurons and sensory neurons (Fig. 1B). The expression pattern of presynaptic genes *chata*, *hacta*, *vachta* and *vachtb* appeared different from that of *chrna*_*3*_ and *chrna*_*2*_*a*. Presynaptic genes, *chrna7* and *ache* were located more ventrally compared to *chrna3* and *chrna2a* (Fig. 2A). We carried out transverse sections of embryos and found *hacta, ache* and *chrna7* expression in the ventral area, while *chrna3* was found in the medial area of the spinal cord (Fig. 2A). These results suggest that the presynaptic cholinergic genes, *ache*, and *chrna7* are expressed in motoneurons while *chrna3* and *chrna2a* are expressed in interneurons. This was confirmed by carrying out fluorescence in situ hybridization (FISH) in a transgenic line that expresses the green fluorescent protein (GFP) under the promoter for the Motor Neuron and Pancreas Homeobox 1 (*mnx1*) gene [*Tg*(*mnx1:GFP*)], which is commonly used to identify motoneurons^31^. We found that *hacta, ache* and *chrna7* expressing cells co-express *mnx1:*GFP, indicating that they are indeed expressed in motoneurons. Interestingly, less than half of *mnx1:*GFP neurons co-express *chrna7*, suggesting that while most motoneurons express *hacta* and *ache, chrna7 *is found only in a subpopulation of motoneurons (Fig. 2D). Conversely, most *chrna3* expressing cells did not co-express *mnx1:*GFP, suggesting that they are expressed mainly in interneurons (Fig. 2B–D). To confirm that *chrna3* is expressed in neurons and not in neural progenitor cells, we carried out FISH and labeled neurons using anti-Hu antibody, a marker for differentiated neurons in zebrafish^32,33^. We found that all *chrna3-*expressing cells were also positive for anti-Hu labeling (Supplementary Fig. 2). Based on the medial location of *chrna3-*expressing neurons along the dorsal–ventral spinal cord (Fig. 2A), we conclude that *chrna3* is indeed expressed in interneurons in the embryo.

In adult zebrafish and rats, there is no evidence for cholinergic descending projections from the brain to the spinal cord but only intraspinal cholinergic innervation^34–36^. However, to test for the possibility of transient cholinergic neurons from the brain that project to the nAChR-expressing interneurons in the spinal cord, we also examined the expression of cholinergic genes in the brain. We found faint ubiquitous expression of *vachtb, ache, chrna3* and *chrna7* transcripts at 22–24 hpf in the embryonic brain (Fig. 1D, F, Supplementary Fig. 1B, D), suggesting that they were not specific to cholinergic neurons. Only *chatb* was expressed in distinct nuclei adjacent to the eyes (Supplementary Fig. 1E), suggesting the presence of the putative oculomotor cholinergic neurons. These results showing a lack of clear presynaptic gene expression in the brain combined with the strong expression of both presynaptic and nAChR subunits in the spinal cord suggest that the nAChRs in the interneurons may detect ACh released from motoneurons during early embryonic development.

### Presynaptic cholinergic genes and *ache* transcripts are decreased in the spinal cord from 3 days

We found that *chata*, *hacta* and *vachtb* are expressed in the brain of 2-day-old embryos (Supplementary Fig. 3). *chata* was expressed in oculomotor (asterisk), secondary gustatory (arrowhead), trigeminal motor nuclei (arrow) and cluster of hindbrain neurons. *hacta* and *vachtb* transcripts were found in midbrain nuclei (brackets). Additionally, *vachtb* transcripts were detected in the habenula (white arrowhead). The expression of cholinergic genes did not differ greatly in 2-day-old spinal cord compared with that in 24 hpf embryos (data not shown).

**Figure 3:**
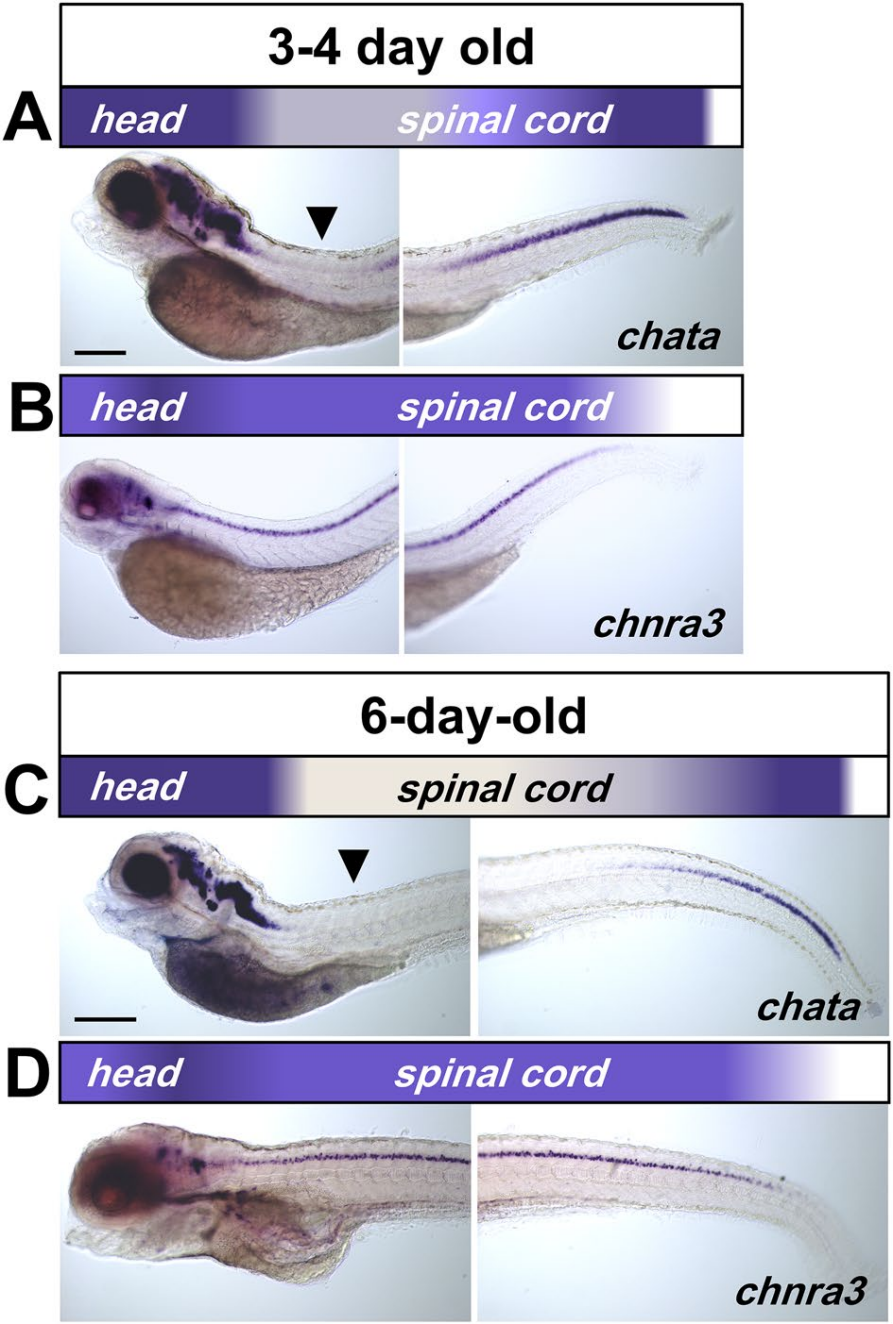
Downregulation of presynaptic cholinergic gene transcript in larval spinal cord. (**A**, **C**) Lateral view of 3–4 and 6-day-old larvae processed by in situ hybridization showing a rostro-caudal gradient for *chata*. Arrowheads point toward reduced transcript levels in rostral spinal cord. (**B**, **D**) *chrna3* remain expressed in a uniform pattern along the entire spinal cord. The rostro-caudal expression pattern of the transcript is represented by the colored gradient bar on top of each image. Note that there is no change in transcript levels in the brain. Magnification is the same for all images in (**A**–**D**). Scale bar: 200 μm.

In 3- and 4-day old embryos more areas in the brain start expressing *chata*, *hacta* and *ache*. However, ISH staining for these genes start to become fainter at the rostral spinal cord (arrowhead) (Fig. 3A, Supplementary Fig. 4A, B). In addition, while *chatb* transcript remained absent in the spinal cord, *vachtb* expression decreased drastically and is barely detectable in the spinal cord by 3 days (Supplementary Fig. 4C, D). By contrast, nAChR subunits α_3_ and α_7_ transcripts remain uniformly highly expressed throughout the spinal cord (Fig. 3B, Supplementary Fig. 4E). These results suggest that from 3 days, there is a decrease of presynaptic cholinergic transcripts and *ache* in a rostral to caudal gradient.

In 6-day-old larvae, ISH staining for *chata*, *vachta*, *hacta* and *ache* in the spinal cord is mainly expressed in the caudal spinal cord (Fig. 3C, Supplementary Fig. 5A–C). Conversely, nAChR subunits α_3_ and α_7_ transcripts remain expressed along the spinal cord (Fig. 3D, Supplementary Fig. 5D). *chata* transcript expression continues to decrease in the rostral spinal cord of 10-day-old larvae and becomes limited to the tip of the tail in juvenile fish (15 days) (Supplementary Fig. 6). These findings suggest that only presynaptic cholinergic genes in the spinal cord undergo the rostral to caudal downregulation during development, which continues until juvenile stages.

### Rostro-caudal gradient of ChAT and VAChT in larvae

**Figure 4:**
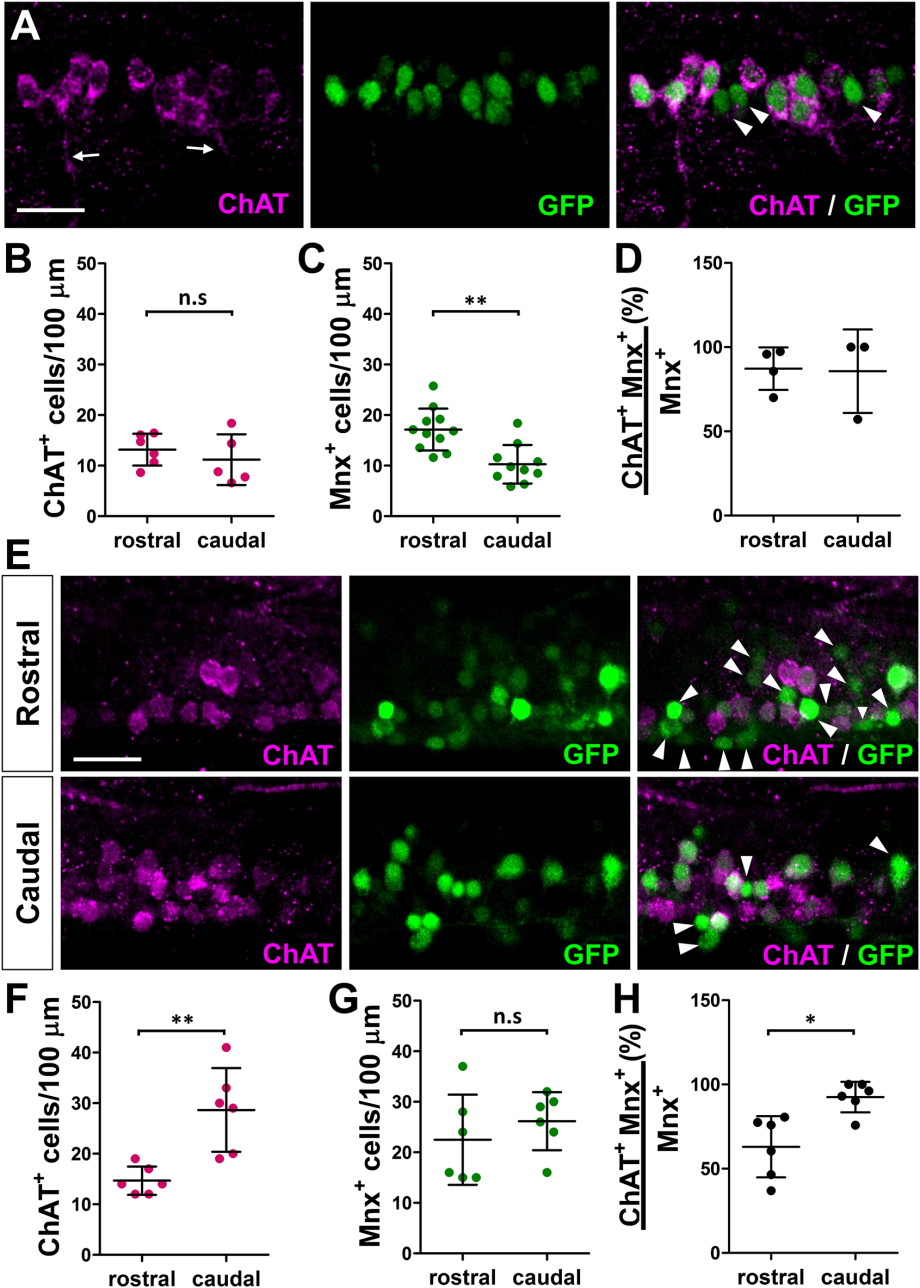
Expression of ChAT in *Tg*(*mnx1:*GFP) spinal motoneurons. (**A**) Confocal images of a lateral view of 24 hpf embryos processed with antibody labeling showing ChAT in MN soma and axons (arrows). Arrowheads point to ChAT^-^ *mnx1:*GFP^+^ cells. (**B**–**D**) Quantification of ChAT and *mnx1:*GFP labeled cells in 24 hpf *Tg*(*mnx1:GFP*) embryos. (**B**) ChAT^+^ and (**C**) *mnx1:*GFP labeled cell numbers in 100 μm segment in rostral and caudal spinal cord of 24 hpf embryos. (N = 10 embryos; 527 *mnx1:*GFP^+^ neurons, 307 ChAT^+^ neurons). (**D**) Percentage of ChAT expressing *mnx1:*GFP neurons in rostral and caudal spinal cord. (N = 7 embryos; 186 *mnx1:*GFP^+^ neurons, 172 ChAT^+^ neurons). (**E**) Confocal images of a lateral view of a 6-day-old larva processed with antibody labeling showing that ChAT^-^ *mnx1:*GFP^+^ cells (arrowheads) are found more in the rostral than caudal spinal cord. (**F–H**) Quantification of ChAT and *mnx1:*GFP labeled cells in 6-day-old *Tg*(*mnx1:GFP*) larvae. (**F**) ChAT^+^ and (**G**) *mnx1:*GFP labeled cell numbers in 100 μm segment in the rostral and caudal spinal cord of 6-day-old *Tg*(*mnx1:GFP*) larvae. (**H**) Percentage of ChAT expressing *mnx1:*GFP neurons in rostral and caudal spinal cord. (N = 6 larvae; 499 *mnx1:*GFP^+^ neurons, 381 ChAT^+^ neurons). All images are oriented with rostral towards the left and dorsal to the top. Scale bar: 20 μm. 2-tailed non-paired Student’s *t* test; n.s.: not significant; *p < 0.05; **p < 0.005.

To verify if the downregulation of presynaptic cholinergic mRNA transcript expression is consistent with a decrease in protein levels, we carried out immunohistochemistry (IHC) for ChAT in the *Tg*(*mnx1:GFP*) transgenic line. First, we found in 24 hpf embryos that while the rostral spinal cord contained higher number of motoneurons (17.14 ± 4.14 vs. 10.27 ± 3.8 l; *p* = 0.0009), there was no difference in the number of ChAT^+^ neurons between the rostral and caudal spinal cord (Fig. 4A–C; 13.17 ± 3.13 vs. 11.19 ± 5.01; *p* = 0.444). In addition, we found that ChAT is expressed in most motoneuron soma and axons in the spinal cord at this stage of development (Fig. 4A, D; 87 ± 17%). In 6-day-old larvae, while the number of motoneurons in the rostral vs. caudal spinal cord was similar (23 ± 8.92 vs. 26 ± 5.74; *p* = 0.417), there was almost twice as many ChAT^+^ neurons in the caudal spinal cord (Fig. 4E–G; 15 ± 2.8 vs. 29 ± 8.26; *p* = 0.0028). In fact, the caudal motoneurons mostly co-expressed ChAT (93 ± 9.1%), as found in 24 hpf embryos. However, the rostral spinal cord showed a decrease in the percentage of motoneurons that co-express ChAT (Fig. 4H; 63 ± 18%; *p* = 0.005). These results show that ChAT protein expression is correlated with its mRNA transcript expression, and suggest that ChAT is downregulated in the rostral spinal cord during larval development.

**Figure 5:**
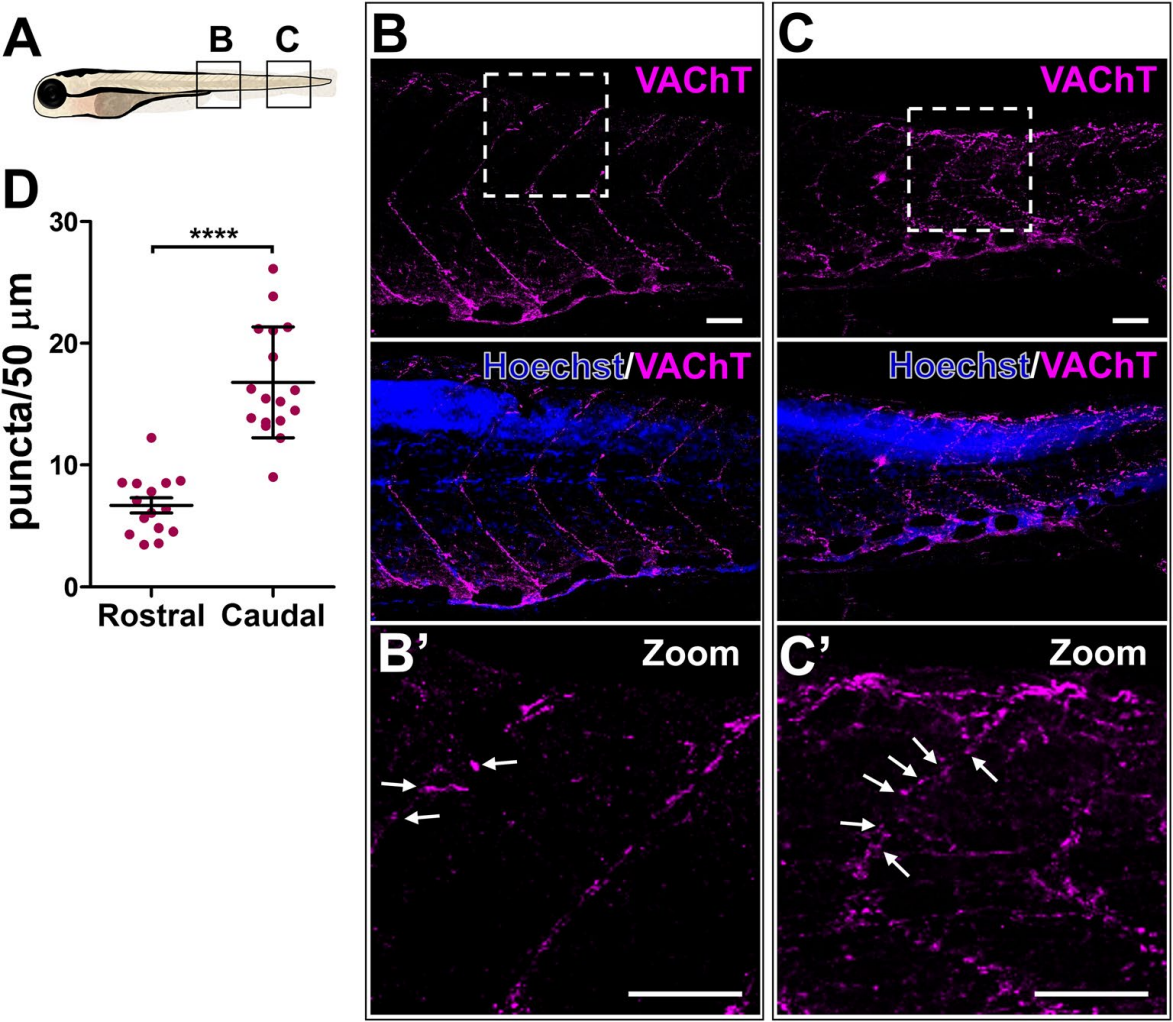
Decreased VAChT expression along the rostral myosepta of 6-day-old larva. (**A**) Schematic representation of the imaged areas in (**B**, **C**). (**B**, **C**) Confocal image of a lateral view of larva showing a rostro-caudal gradient of VAChT puncta in MN axons projecting along the dorsal myosepta. Nuclei are labeled by Hoechst (blue). (**B**′, **C**′) Zoomed in images of the boxed areas in (**B**, **C**). White arrows point toward VAChT positive signals along the myosepta. Scale bars: 50 μm. (**D**) Quantification of VAChT puncta in the dorsal myosepta. N = 4 larvae (15 rostral axons, 17 caudal axons). 2-tailed non-paired Student’s *t* test; ****p < 0.0001.

To see whether the difference in the number of ChAT^+^ neurons in the spinal cord affects the number of putative ACh release sites, we next carried out IHC for VAChT, which functions to package ACh into vesicles at its release sites. We quantified the number of VAChT puncta at the neuromuscular junctions in the rostral vs. caudal myosepta, which have been shown to contain high levels of nAChRs^37^. Again, we found that there was a significantly lower number of VAChT puncta in the rostral compared to caudal myosepta (Fig. 5; 6.68 ± 2.4 vs. 16.788 ± 4.56; *p* = 2.67391E−08), suggesting a rostro-caudal gradient of VAChT in the spinal motoneuron projections to the myosepta in 6-day-old larvae. This gradient is consistent with *vachta* ISH results (Supplementary Fig. 5A) further providing evidence that downregulation of presynaptic cholinergic component mRNA expression does indeed result in decreased protein levels.

### Reduced AChE activity in the rostral spinal cord

**Figure 6:**
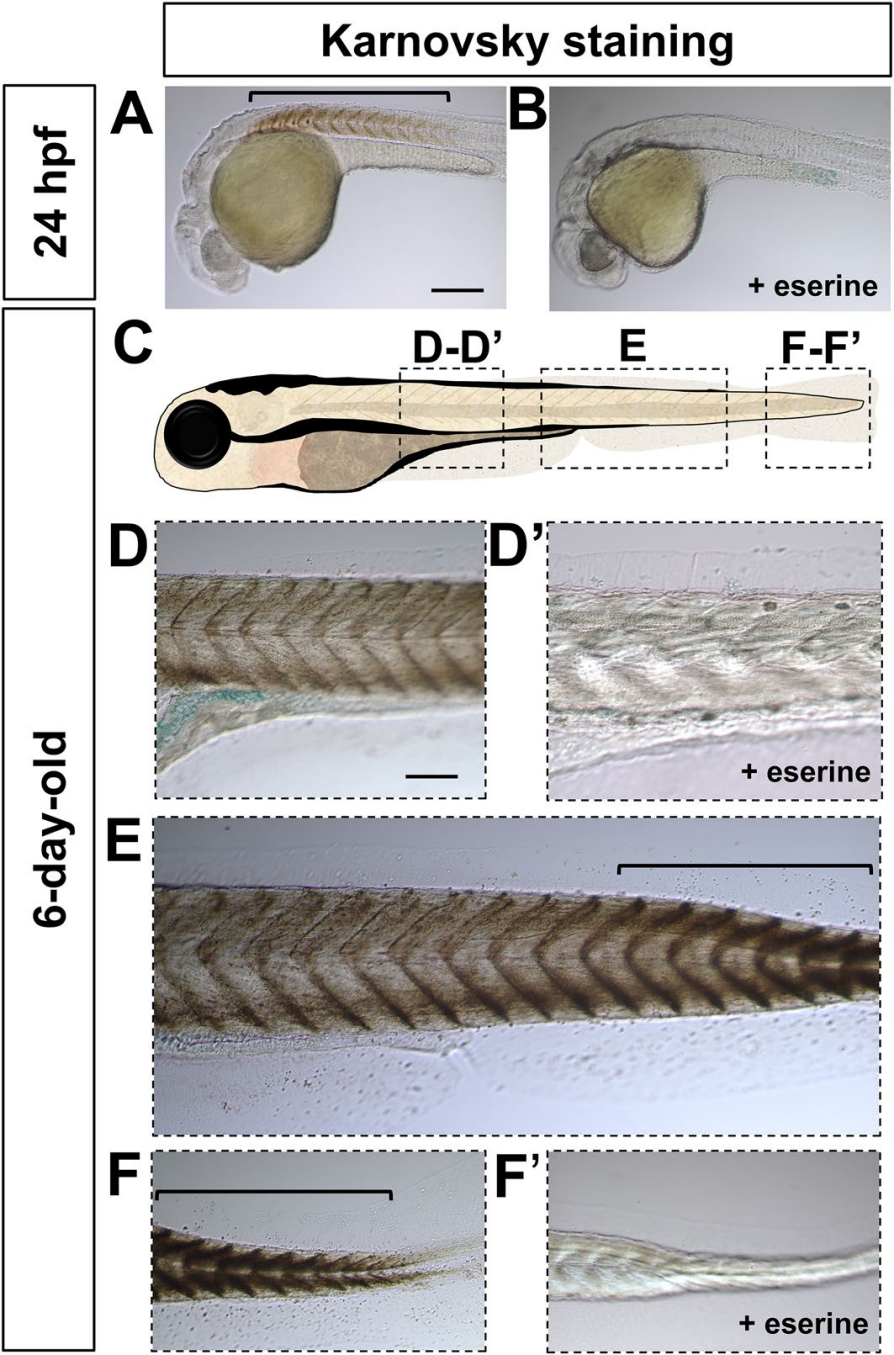
Spatiotemporal Acetylcholinesterase enzymatic activity pattern in the spinal cord during early development. (**A**, **B**) Lateral view of 24 hpf embryos processed with Karnovsky staining showing Acetylcholinesterase enzymatic activity (bracket) in the absence (**A**), but not in presence (**B**) of eserine, an AChE inhibitor. (**C**) Schematic representation of the imaged areas in (**D**–**F**′). Lateral view of 6-day-old larvae showing a rostro-caudal gradient of AChE activity revealed by Karnovsky staining (**D**–**F**′). (**D**, **D′**) Anterior trunk in the absence (**D**) or presence (**D**′) of eserine. (**E**) Mid trunk in the absence of eserine. (**F**, **F′**) Tip of the tail in the absence (**F**) or presence (**F**′) of eserine. Brackets indicate high levels of Karnovsky staining in (**E**, **F**). Magnification is the same for all images in (**A**, **B**) and (**D**–**F**′). Scale bar: 200 μm.

Another cholinergic gene that was decreased in the rostral spinal cord in larvae is *ache*. We carried out Karnovsky staining to assay for esterase activity^38^. First, we performed this in 24 hpf embryos where we found stronger staining in the rostral compared with the caudal spinal cord (Fig. 6A), which is consistent with *ache* expression pattern (Fig. 1D). This staining was completely lost when embryos are treated with eserine (Fig. 6B), an esterase inhibitor, showing the specificity of the staining. In 6-day-old larvae, we found stronger staining in the caudal compared with the rostral spinal cord (Fig. 6C–F), which is lost when treated with eserine (Fig. 6D′, F′). This result is consistent with the rostro-caudal gradient in *ache* expression (Supplementary Fig. 5C), and indicates that the level of AChE enzymatic activity is determined by *ache* transcript expression.

### Downregulation of cholinergic signaling is not due to neurotransmitter switch in motoneurons

Downregulation of presynaptic cholinergic gene expression during development may be explained by a change in the neurotransmitter type utilized by motoneurons in the spinal cord during later stages of development. Indeed, studies have shown co-release of ACh and glutamate in motoneurons in the perinatal and adult mouse^39,40^ and in the adult zebrafish^36^. Only the *vesicular glutamate transporter 2 *(*vglut2*)*,* and not *vglut1* is expressed in the zebrafish spinal cord in embryo and larvae^41^. We assayed whether motoneurons co-express *vglut2* by analyzing the *Tg*(*mnx1:GFP;vglut2:DsRed*) double transgenic line. Our results show that in 24 hpf embryos *vglut2:*DsRed neurons are located dorsal to the *mnx1:*GFP neurons (Supplementary Fig. 7A), consistent with a previous study suggesting that only interneurons utilize glutamate as their neurotransmitter in the zebrafish embryo^41^. In 6-day-old larvae, interneurons continue expressing *vglut2*, while less than 5% of *mnx1:*GFP^+^ neurons co-express *vglut2:*DsRed (Supplementary Fig. 7B, C; 2.9 ± 2.5%). Together, these findings suggest that the decrease in cholinergic phenotype in motoneurons is not due to the acquisition of the glutamatergic phenotype.

**Figure 7:**
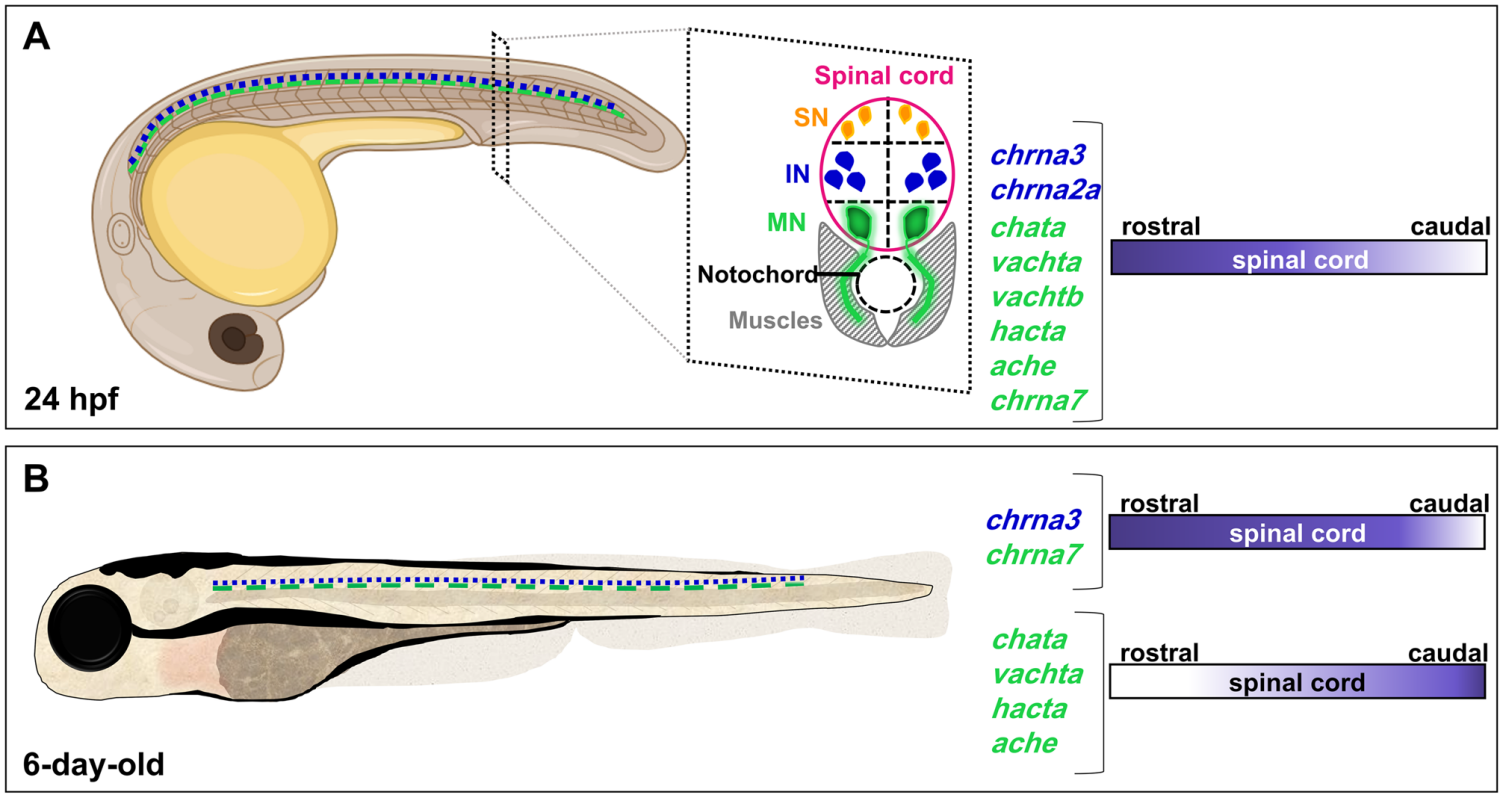
Spatiotemporal cholinergic gene map in embryonic and larval zebrafish. (**A**, **B**) Illustration of 24 hpf embryo (**A**) and 6-day-old larva (**B**) summarizing the cholinergic genes expressed in spinal motoneurons (green) and interneurons (blue). The rostro-caudal expression pattern of the transcripts is represented by the colored gradients on the right. Abbreviations:  sensory neurons (SN), interneurons (IN), motoneurons (MN).

## Discussion

We carried out a detailed spatiotemporal analysis of one of the most ancient signaling systems in living forms, the cholinergic system, which can be perturbed by external sources including nicotine and neonicotinoid pesticides. This spatiotemporal map revealed that while 1-day-old embryos express the full machinery for ACh release in motoneurons and interneurons express different subunits for the nicotinic ACh receptors, there is an unexpected decrease in presynaptic cholinergic components in the rostral spinal cord during development (Fig. 7). The downregulation of presynaptic cholinergic transcripts was validated by the reduction in ChAT-expressing neurons, as well as the number of VAChT puncta along the myosepta in the rostral versus caudal spinal cord. Finally, acetylcholinesterase activity staining confirmed that the rostral to caudal gradient of *ache* transcript translates to reduced AChE function in the rostral spinal cord.

In the spinal cord, there is a rostral to caudal delay in the differentiation of neurons during development. In other words, rostral neurons are more mature than caudal neurons^30^. The higher number of ChAT^+^ neurons, VAChT^+^ protein puncta and AChE activity in the caudal compared with the rostral spinal cord suggest that there is a dynamic downregulation of cholinergic neurons during development. It is possible that the downregulation of cholinergic neurons may be due to programmed cell death (PCD) as around 40% of cells from the lateral motor column undergo PCD in the chick embryo^42^. However, studies in rat, mouse or chick show conflicting results on whether there is indeed a rostro-caudal gradient of PCD^42,43^. Furthermore, PCD was not observed in motoneurons^44^ and appears to mainly occur in sensory neurons in the zebrafish spinal cord during early development^45^. Regardless, even if the reduction in ChAT^+^ neurons in the rostral spinal cord may be partly due to PCD, this cannot fully explain the lower levels of mRNA transcript staining, which becomes even more difficult to detect in juvenile larvae (15-days-old), except at the tip of the tail. As the staining is maintained at high levels in the brain throughout development, the difference in staining intensity is unlikely due to technical issues.

Studies in perinatal and adult mouse have shown that spinal motoneurons exhibit developmental plasticity by co-releasing acetylcholine and glutamate in the CNS^39,40^. Recent study in adult zebrafish found evidence that motoneurons co-release glutamate at the neuromuscular junction depending on their physiological and pathophysiological conditions to regulate motor behaviors^36^. We hypothesized that the reduction in cholinergic neurons in the rostral spinal cord may be due to a transition to a glutamatergic neurotransmitter phenotype. However, as less than 5% of *mnx1:*GFP^+^ neurons appear to be glutamatergic, this cannot fully account for the ~ 30% reduction in ChAT-expressing motoneurons in the rostral spinal cord.

One characteristic of ACh is that it functions as both a neurotransmitter, mediating fast synaptic transmission and a neuromodulator, to alter neuronal excitability and/or coordinate firing of groups of neurons^46^. It functions as a neurotransmitter at the neuromuscular junction (NMJ) between the motoneuron and muscles. In the brain, evidence of ACh function as a neurotransmitter is scarce, instead, it is released at non-synaptic sites through exocytosis and/or volume transmission and is believed to function as a neuromodulator^46,47^. In vertebrates including rodents and chick, cholinergic signaling mediates the emergence of spontaneous neuronal activity (SNA) in the embryonic spinal circuit^11–13^. However, this effect is transitory as ChAT mutant mice recover their SNA, albeit at reduced levels compared with wild-type animals^12^. We propose that in embryonic stages, ACh released by MNs functions as a neuromodulator for the synchronized activation of interneurons. Indeed, ACh released from motoneurons is necessary for the activation of GABAergic and glutamatergic interneurons in the embryonic mice spinal cord^48^. Later, as spinal neurons mature in a rostral to caudal sequence, ACh may be mainly released by motoneurons at the neuromuscular junction and functions as a neurotransmitter in regulating fast synaptic currents, necessary for the precise control of locomotion.

The relationship between gene expression patterns with mRNA levels, decay, stability or protein levels appear complex as it varies depending on many factors including the developmental stage and cellular location, as well as function of the protein^49,50^. An interesting hypothesis to explore in future studies would be to test whether the high and low levels of presynaptic mRNA transcript result in either neuromodulation or neurotransmitter functions during cholinergic signaling, respectively.

The emergence of spinal SNA in 16 h to 1-day-old zebrafish embryo^51,52^, when cholinergic genes start being expressed, suggests that cholinergic signaling plays a role in the generation of SNA and spinal circuit formation, as have been described in other vertebrate animals^11–13^. However, previous studies have concluded that this may not be the case as they failed to observe an effect on embryonic spinal neuron activity upon application of cholinergic antagonists in zebrafish^53,54^. Our work unequivocally shows that the cholinergic machinery is present in 1-day-old embryos, which is then downregulated during development. These results strongly suggest that cholinergic signaling may indeed play a key conserved role during embryonic CNS development.

Together, these findings showing the complexity, dynamics and conservation of zebrafish spinal cholinergic neurons during development, warrant further investigation on the role of cholinergic signaling in SNA and circuit formation in the zebrafish embryo.

## Materials and methods

### Zebrafish embryos

All animals were maintained at 28 °C in a 14/10 h light/dark cycle at the IBPS core facility according to established procedures. Wild-type AB strain, *Tg*(*mnx1:GFP*)^31^*,* and *Tg*(*vglut2:DsRed*)^55^ lines were used in this study. Maintenance of zebrafish stocks and experiments on larvae were carried out in accordance with the “Comité d'éthique Charles Darwin” (APAFIS#15,909–2,018,070,912,072,530 v5). The fish facility has been approved by the French “Service for animal protection and health” (A-75-05-25). Zebrafish embryos were collected and allowed to develop at 28.5 °C. To prevent pigment formation, 0.2 mM phenylthiourea (PTU) was added to the fish water starting 24 h post fertilization (hpf).

### RNA colorimetric and fluorescent In-situ hybridization (ISH)

Colorimetric ISH on whole-mount zebrafish embryos and larvae was performed as described in^56^. Plasmids used for antisense RNA probes preparation were described in^47^. Restriction enzymes and RNA polymerases used to synthesize the probes were: *vachta* (BamH1/SP6), *vachtb* (EcoRI/T7), *chata* (Not1/Sp6), *chatb* (Not1/Sp6), *hacta* (Not1/SP6), *chrna2a* (Not1/Sp6), *chrna3* (BamH1/T7), *chrna7* (Not1/Sp6), and *ache* (HindIII/T7). Briefly, probes were labeled with UTP-digoxigenin and incubated with embryos overnight in 50% formamide-containing hybridization solution. Probes were then detected using alkaline phosphatase conjugated anti-Digoxigenin antibodies (Roche) and visualized by 5-bromo-4-chloro-3-indolyl-phosphate (BCIP) and 4-nitro blue tetrazolium (NBT) staining. When the desired labeling intensity is reached, embryos/larvae were washed extensively and stored in PBS at 4 °C until imaging.

Fluorescent in situ hybridization (FISH) was performed on 24 hpf embryos as described in^47^. Briefly, UTP-digoxigenin labeled probes were incubated with embryos overnight in 50% formamide-containing hybridization solution. Probes were then detected using horseradish peroxidase conjugated anti-Digoxigenin antibody (Roche) in Maleic acid blocking buffer (150 mM Maleic acid, pH 7.5 & 100 mM NaCl). To amplify the signal, homemade tyramide-FITC was used as described in^57^. Embryos were incubated with tyramide-FITC (1:250) in TSA reaction buffer (100 mM borate pH 8.5, 0.1% tween-20, 2% dextran sulfate, 0.003% H_2_O_2_, and 200 µg/ml 4-iodophenol) for 30–45 min in the dark. The reaction was then stopped by abundant washes of 0.1% Tween-20 in PBS (PBT). Embryos were incubated overnight at 4 °C in PBS, 0.1% Triton, 10% sheep serum with rabbit anti-GFP antibody (Thermofisher) or mouse anti-HuC/HuD antibody (Life Technologies) to detect GFP or neurons, respectively. Embryos were washed in PBT and incubated at 4 °C overnight with goat anti-rabbit Alexa594 antibody or goat anti-mouse Alexa555 antibody (Thermofisher), respectively. Finally, embryos were abundantly washed and stored in PBS at 4 °C in the dark until imaging.

### Immunohistochemistry

Immunohistochemistry (IHC) was performed either on whole-mount or sectioned larvae as described in^58^, with some modifications. Larvae were fixed for 1.5 h at 4 °C in 4% paraformaldehyde, then washed (3 × 5 min) in PBS with 0.2% Triton X-100 (PBST). Samples were then blocked on shaker for 3 h in PBS with 1% BSA, 1% DMSO, 5% Normal Sheep Serum, and 0.2% Triton X-100. Larvae were then incubated with rabbit polyclonal anti-VAChT antibody (1:500, Synaptic systems) in PBST with 1% BSA, overnight at 4 °C on a shaker. Samples were washed in PBST, and incubated with anti-rabbit Alexa594 for 5 h, then Hoechst (Thermofisher) for 30 min at room temperature on a shaker. After several PBST washes, larvae were stored in PBS at 4 °C until imaging. ChAT IHC was performed on whole-mount embryos and larvae. Briefly, embryos/larvae were fixed for 5 h at 4 °C in 4% paraformaldehyde, washed with PBS, and then permeabilized with 0.8% PBST for 1 h on shaker. Samples were then incubated with goat anti-ChAT (1:100, Millipore) for 72 h at 4 °C on shaker. After 3 washes of PBS, samples were incubated with anti-goat Alexa594 overnight at 4 °C on shaker. After several PBST washes, larvae were stored in PBS at 4 °C until imaging.

### Detection of AChE enzymatic activity

AChE activity was detected using a method adapted from Karnovsky and Roots^38^. Briefly, larvae were fixed for 7 h in BT-Fix^59^ at room temperature, then washed in PBS with 0.1% Triton X-100. Larvae were then incubated 4–5 h in 60 mM sodium acetate buffer (pH 6.4), 5 mM sodium citrate, 4.7 mM CuSO_4_, 0.5 mM K_3_(Fe(CN)_6_), and 1.7 mM acetylthiocholine iodide. When the desired labeling intensity was reached, larvae were washed extensively and stored in PBS at 4 °C until imaging. To check for the specificity of the staining, acetylcholinesterase inhibitor eserine was used at a final concentration of 10^–4^ M.

### Sectioning and microscopy

To prepare sections, embryos processed with ISH or IHC were embedded in 4% low-melt agarose prepared in PBS and sectioned at 35 µm (transverse) or 50 µm (sagittal) using a vibratome (LEICA VT1000S). ISH sections were then mounted in Mowiol (Sigma-Aldrich) on glass slides, covered with coverslips and stored at 4 °C in the dark until imaging.

Embryos/larvae processed with ISH or Karnovsky staining were transferred to a glass microscope slide with single cavity well containing 50% glycerol. Bright-field images were acquired on Nikon Eclipse E800 microscope equipped with a Nikon DXM1200 digital camera using Lucia G software. The same system was used to image slides with sections.

Larvae processed with FISH and IHC were mounted laterally in a drop of 2% low-melt agarose in medium petri dishes, then covered with PBS. Fluorescent Z-stack images were captured at 0.5–3 μm increments with a Leica SP5 confocal microscope using a 20X water immersion objective. Acquisition parameters were identical when imaging rostral and caudal areas of the same fish.

### Fluorescent in situ hybridization and immunohistochemistry analysis

Quantifications of *hacta*^+^/*mnx1*:GFP*, chrna3*^+^/*mnx1*:GFP*, ache*^+^/*mnx1*:GFP*, chrna7*^+^/*mnx1*:GFP*, chrna3*^+^/HuC, ChAT^+^/*mnx1*:GFP neurons and VAChT puncta were performed manually using ImageJ. VAChT analysis was performed on axon branches of the rostral and caudal motoneurons along the dorsal myosepta. For each axon, the length of the myosepta was measured and VAChT^+^ puncta were counted. Labeled puncta were defined as areas containing at least four attached pixels. At least three rostral and three caudal myosepta segments were analyzed per fish.

### Statistical analysis

Results are represented as mean ± standard deviation (SD). Differences among groups were analyzed with GraphPad Prism 6.0 software (GraphPad Software Inc., San Diego USA) using a Student’s *t* test.

## Supplementary information

Supplementary Information.
