# Author Correction: Dynamic regulation of the cholinergic system in the spinal central nervous system

**DOI:** 10.1038/s41598-021-99492-6

**Published:** 2021-10-04

**Authors:** Mohamad Rima, Yara Lattouf, Maroun Abi Younes, Erika Bullier, Pascal Legendre, Jean-Marie Mangin, Elim Hong

**Affiliations:** 1 INSERM, CNRS, Neurosciences Paris Seine - Institut de Biologie Paris Seine (NPS - IBPS), Sorbonne Université, 75005 Paris, France; 2 Present Address: Institut de Génétique et de Biologie Moléculaire et Cellulaire (IGBMC), INSERM, CNRS, Université de Strasbourg, 67400 Illkirch, France

Correction to: *Scientific Reports* 10.1038/s41598-020-72524-3, published online 18 September 2020

The original version of this Article contained errors in the spelling of the authors Mohamad Rima, Yara Lattouf, Maroun Abi Younes, Erika Bullier, Pascal Legendre, Jean-Marie Mangin & Elim Hong which were incorrectly given as M. Rima, Y. Lattouf, M. Abi Younes, E. Bullier, P. Legendre, J. M. Mangin & E. Hong.

The original Article and accompanying Supplementary Information file have been corrected.

