# Supplementary material for: Dynamic regulation of the cholinergic system in the spinal central nervous system
Source: Sci Rep. 2020 Sep 18;10:15338. doi: 10.1038/s41598-020-72524-3 (PMC7501295; doi:10.1038/s41598-020-72524-3)
Supplement: Supplementary file 1 — Supplementary Information. [file 41598_2020_72524_MOESM1_ESM.pdf]

Supplementary Figures

Dynamic regulation of the cholinergic system in the spinal central nervous system

Mohamad Rima[1,2], Yara Lattouf[1,3], Maroun Abi Younes[1,3], Erika Bullier[1], Pascal Legendre[1], Jean-Marie Mangin[1], Elim Hong[1*]

[1] Sorbonne Université, INSERM, CNRS, Neurosciences Paris Seine - Institut de Biologie Paris Seine (NPS - IBPS), 75005 Paris, France

[2] current address : Institut de Génétique et de Biologie Moléculaire et Cellulaire (IGBMC), INSERM, CNRS, Université de Strasbourg, 67400 Illkirch, France

[3] equal contribution

[*] Corresponding author

Correspondence to elim.hong@inserm.fr

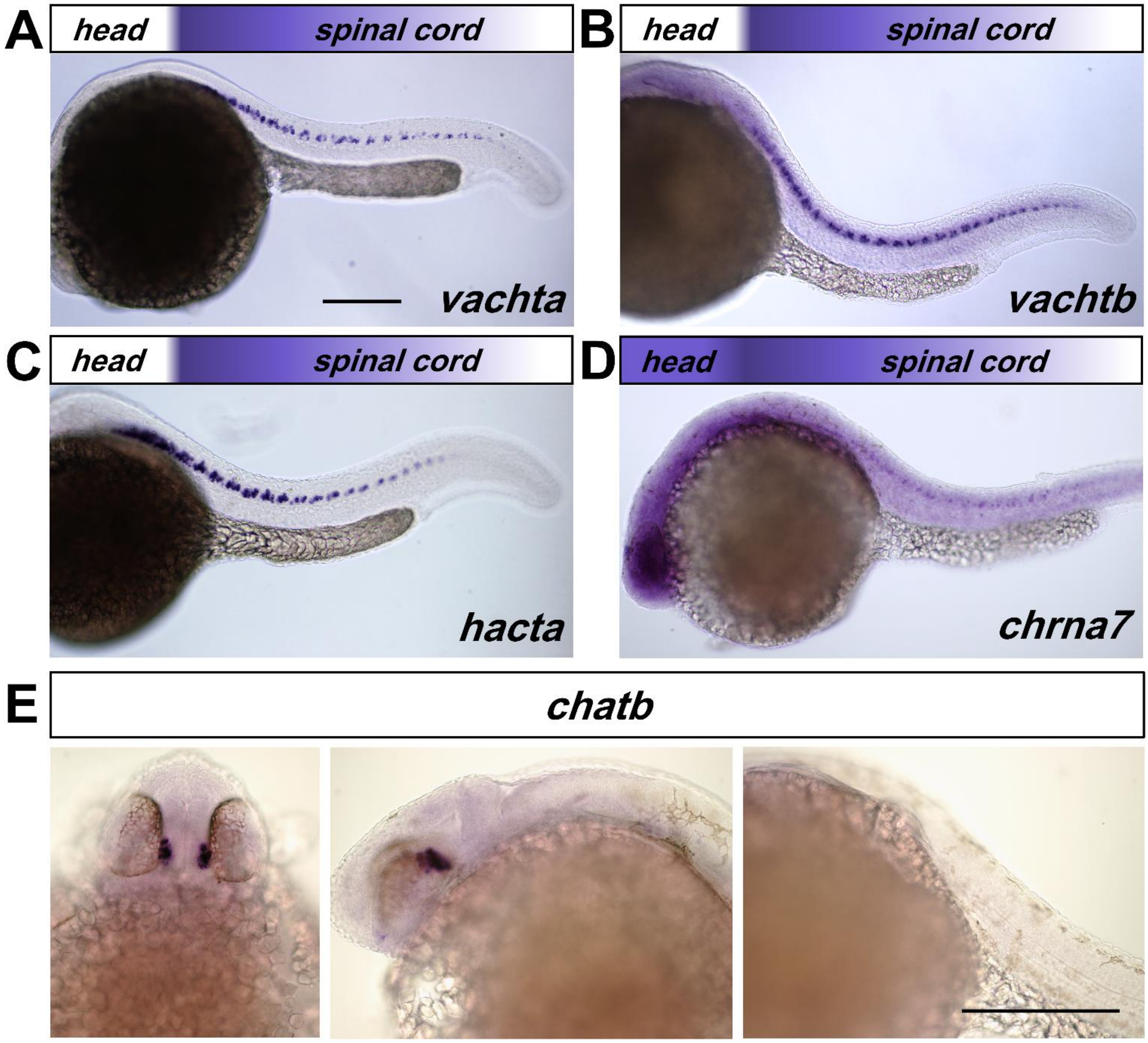

Supplementary Figure 1. Expression pattern of cholinergic markers in the 22-24 hpf embryonic spinal cord. **(A-D)** Lateral view of 22-24 hpf zebrafish embryos processed by *in situ* hybridization showing presynaptic cholinergic transcripts *vachta* (A), *vachtb* (B), and *hacta* (C) in spinal neurons. nAChRs subunit transcript for *chrna7* (D) is expressed in spinal neurons and ubiquitously in the brain. The rostro-caudal expression pattern of the transcript is represented by the colored gradient bar on top of each image. Magnification is the same for all images in (A-D). Scale bar: 200 μm. **(E)** Dorsal (left panel) and lateral (middle, and right panels) view of 22-24 hpf zebrafish embryos processed by *in situ* hybridization showing presynaptic cholinergic transcript *chatb* in the putative oculomotor neurons, but not in the spinal cord. Magnification is the same for all images in (E). Scale bar: 200 μm.

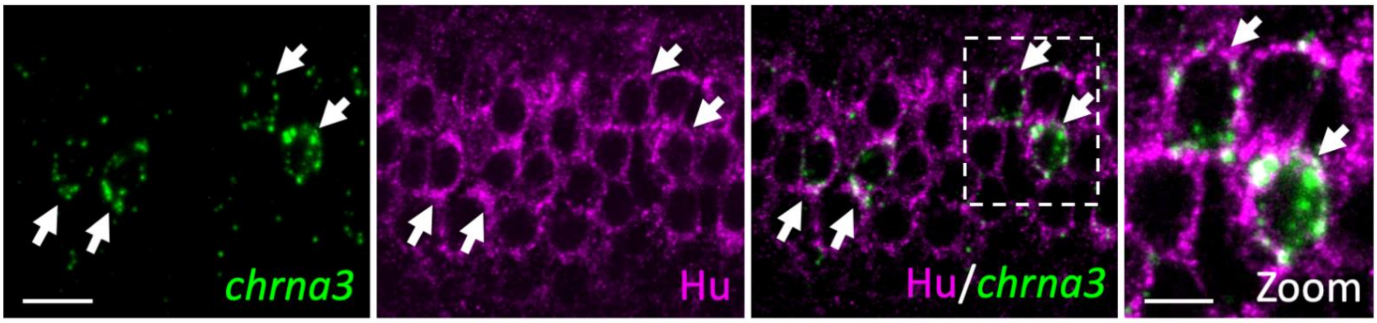

Supplementary Figure 2. Nicotinic acetylcholine receptor subunit *chrna3* is expressed in interneurons. Lateral view of a 24 hpf zebrafish embryo processed by fluorescent *in situ* hybridization for *chrna3* (green) followed by anti-Hu labeling (magenta) shows co-localization of the two signals (white arrows) in 100% of the *chrna3*$^+$ cells. Far right panel (Zoom) shows a close-up image of the white boxed region in the third panel. N = 9 embryos (197 *chrna3*$^+$ cells). Scale bar: 10 μm. Scale bar (Zoom): 5 μm.

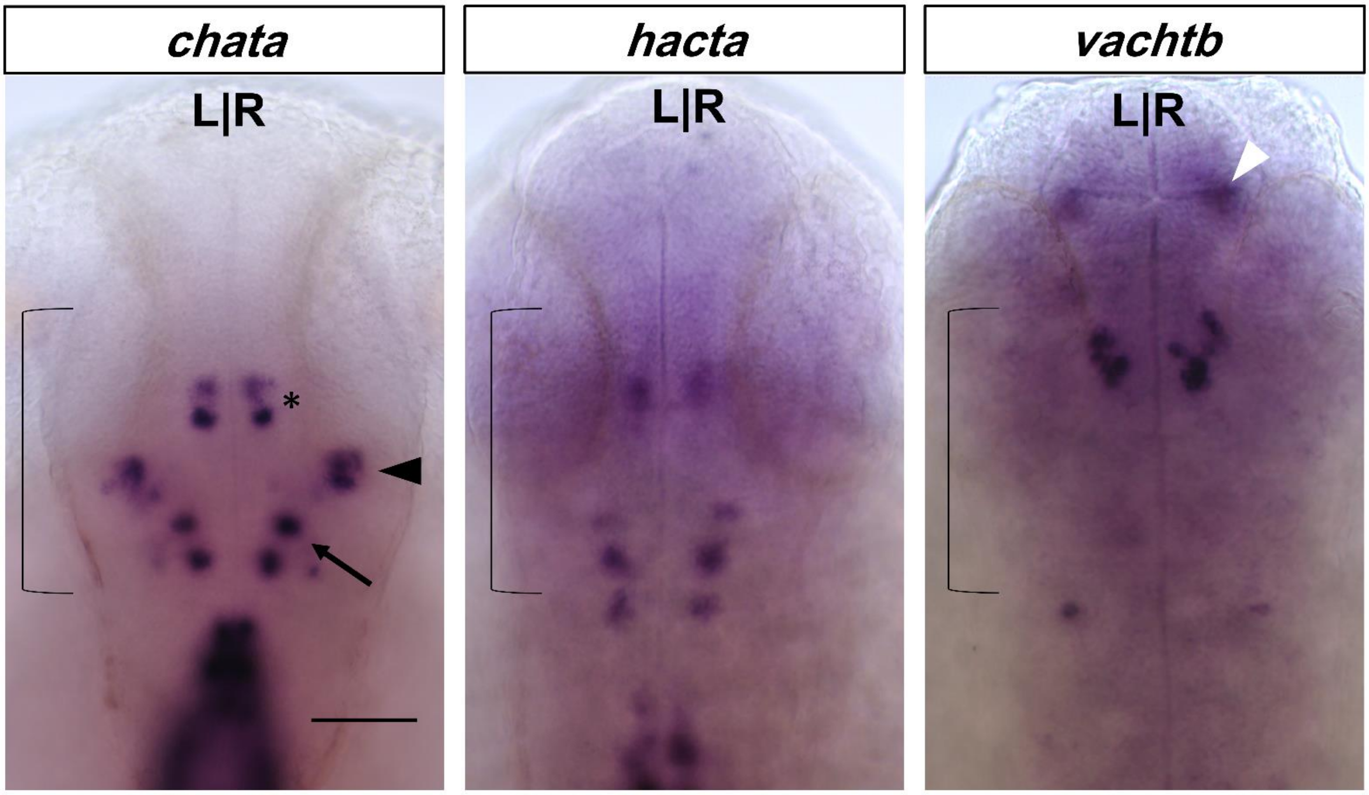

Supplementary Figure 3. Cholinergic gene expression in the brain. Dorsal view of 2-day-old embryos processed by *in situ* hybridization showing *chata*, *hacta* and *vachtb* transcripts bilaterally in the midbrain (brackets). *chata* is expressed in the oculomotor (asterisk), secondary gustatory (arrowhead) and trigeminal motor (arrow) nuclei. *vachtb* is detected symmetrically in the habenula (white arrowhead) at this stage. **L**: left; **R:** right. Scale bar: 200 µm.

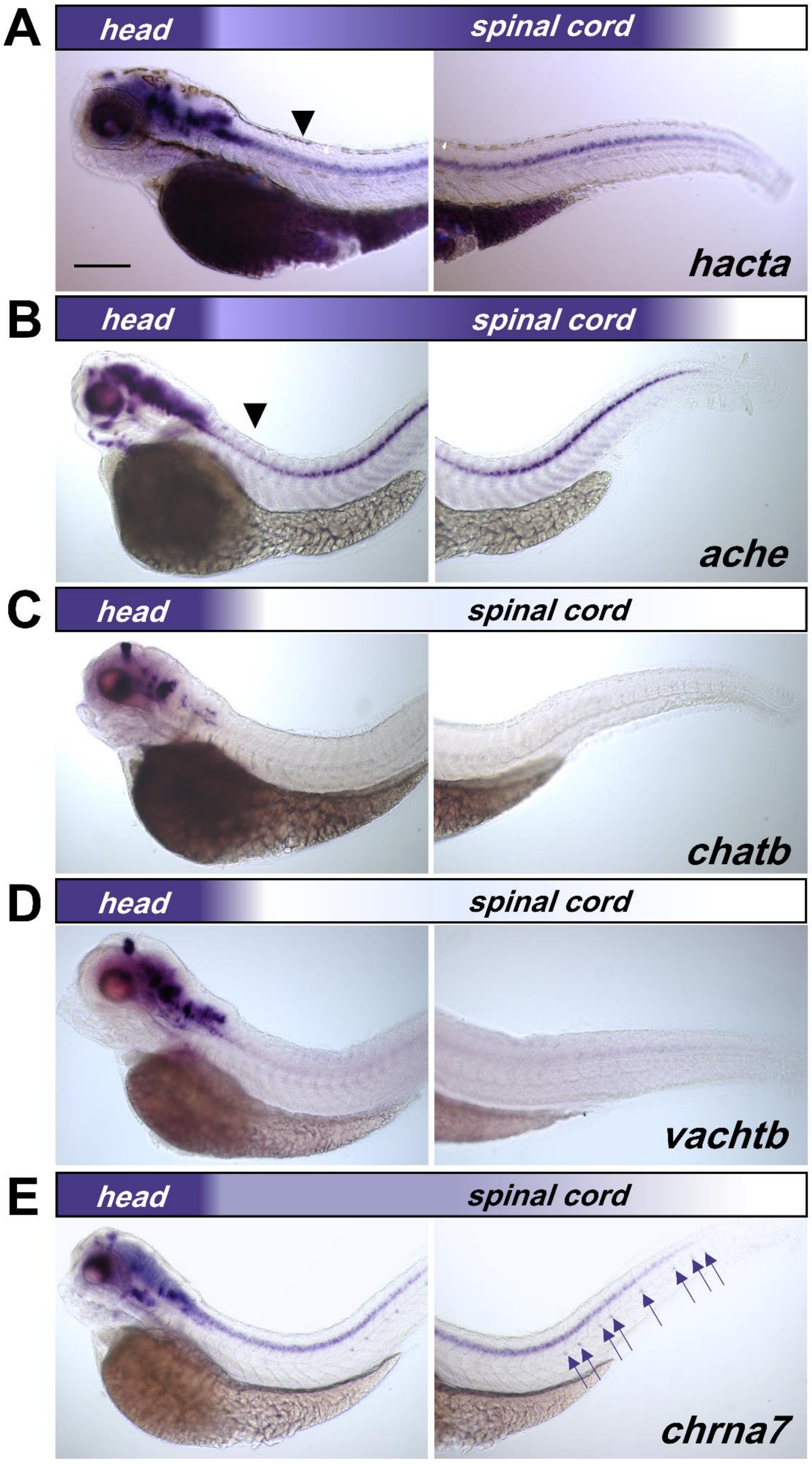

A
head
spinal cord
hacta
B
head
spinal cord
ache
C
head
spinal cord
chatb
D
head
spinal cord
vachtb
E
head
spinal cord
chrna7

Supplementary Figure 4. Downregulation of presynaptic cholinergic gene expression in the spinal cord of 3-day-old embryos. **(A-E)** Lateral view of embryos processed by *in situ* hybridization showing rostro-caudal gradient for *hacta* (A), and *ache* (B). These transcripts remain highly expressed in the brain. *chatb* (C) and *vachtb* (D) are only expressed in the brain. Transcript for the nAChRs subunit *chrna7* (E) remain expressed all along the spinal cord and is also found in the brain and neuromasts (arrows). The rostro-caudal expression pattern of the transcript is represented by the colored gradient bar on top of each image. Magnification is the same for all images in (A-E). Scale bar: 200 μm.

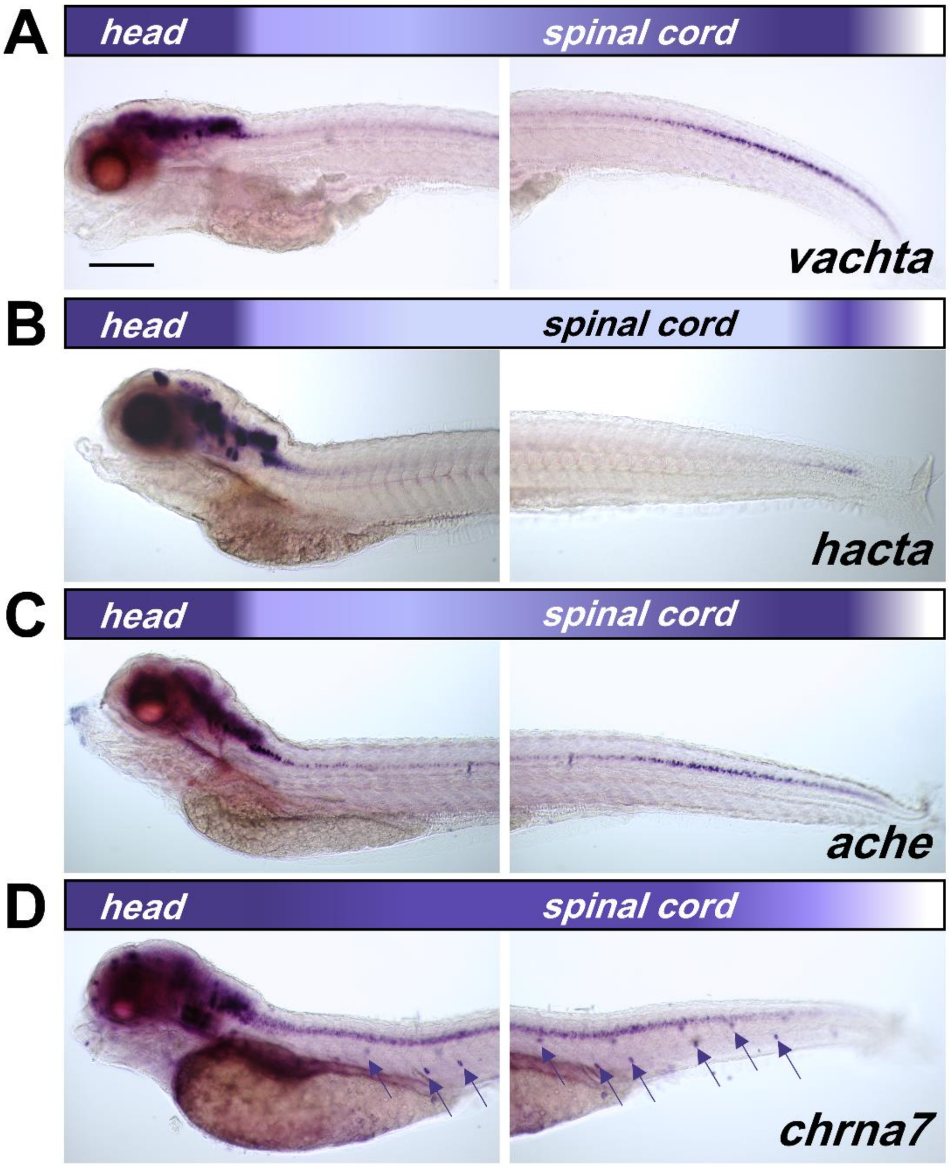

Supplementary Figure 5. Progressive downregulation of presynaptic cholinergic gene expression in 6-day-old larvae. **(A-D)** Lateral view of zebrafish larvae processed by *in situ* hybridization showing the rostro-caudal expression gradient of cholinergic transcripts *vachta* (A), *hacta* (B), and *ache* (C). Note the strong expression of the transcripts in the brain. nAChRs subunit transcript for *chrna7* (D) is also found in the brain, in neuromasts (arrows) and remain uniformly expressed all along the spinal cord. The rostro-caudal expression pattern of the transcript is represented by the colored gradient bar on top of each image. Magnification is the same for all images in (A-D). Scale bar: 200 µm.

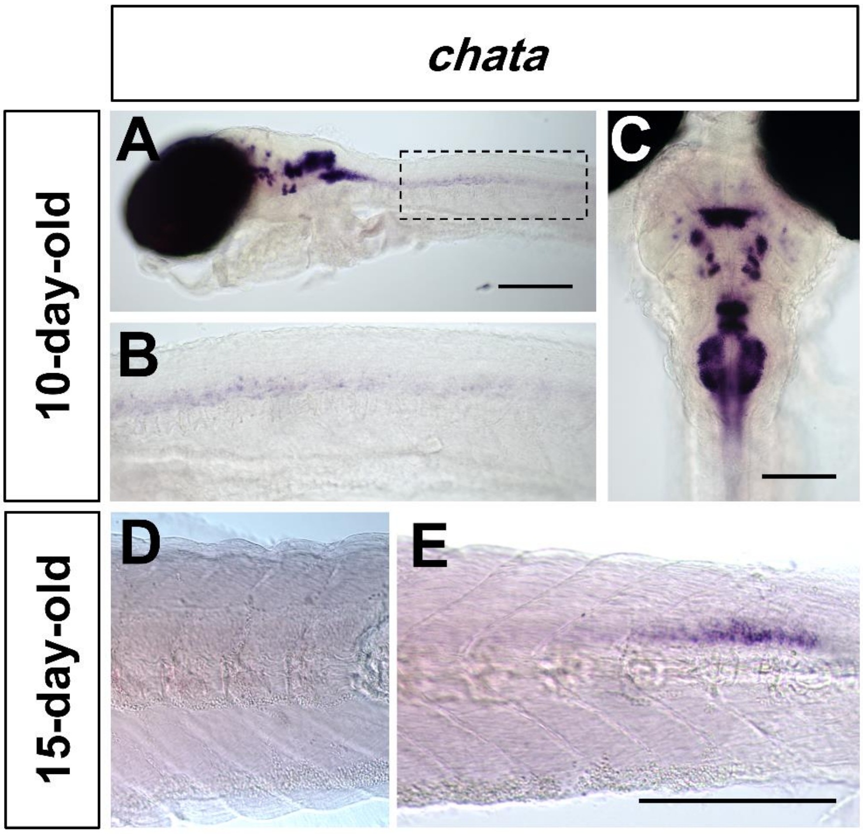

Supplementary Figure 6. Low levels of *chata* expression in the spinal cord persists in 15-day-old larvae. **(A, B)** Lateral and **(C)** dorsal view of a 10-day-old larva processed by *in situ* hybridization showing the decrease in *chata* expression at the rostral spinal cord (A, B), while the transcript remains highly expressed in the brain (C). Panel (B) shows a close-up image of the boxed region in panel (A). Scale bar: 200 μm. **(D)** Lateral view of the rostral spinal cord and **(E)** the tip of the tail of 15-day-old larva processed by *in situ* hybridization showing very faint *chata* expression at the rostral spinal cord, while it remains present at the tip of the tail. Magnification is the same for images in (D, E). Scale bar: 200 μm.

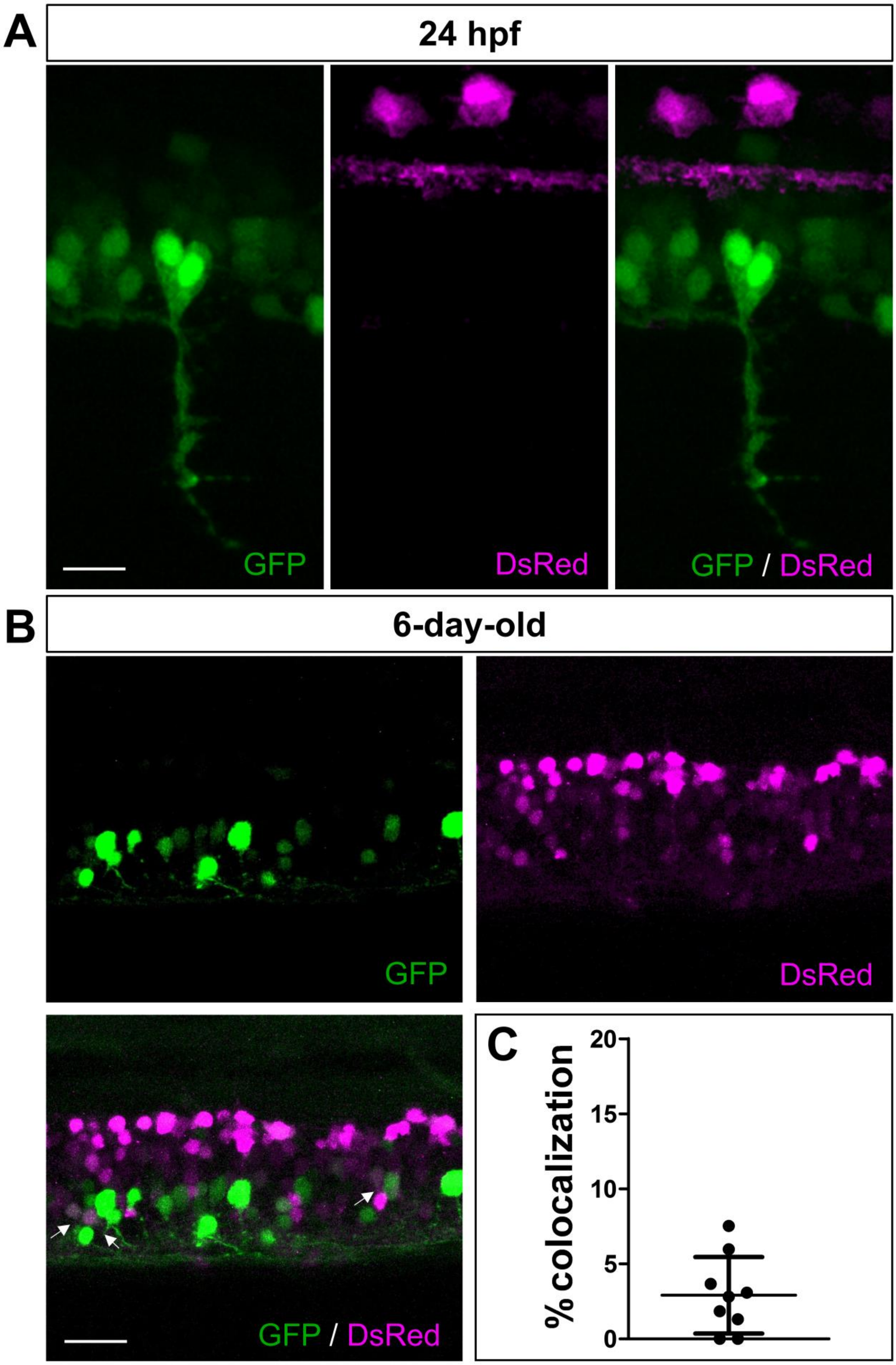
A
24 hpf
GFP
DsRed
GFP / DsRed
B
6-day-old
GFP
DsRed
GFP / DsRed
C
% colocalization
20
15
10
5
0

Supplementary Figure 7. Most *mnx1:*GFP neurons are not glutamatergic in the 6-day-old larvae. **(A-B)** Confocal images of the spinal cord in *Tg(mnx1:GFP;vglut2:DsRed)* line showing no or very few cells co-expressing *mnx1:*GFP (green) and *vglut2:*DsRed (magenta) in 24 hpf embryo (A) or 6-day-old larva (B), respectively. Scale bar: 15 µm in (A) and 20 µm in (B). **(C)** Percentage of *mnx1:*GFP*;vglut2:*DsRed cells in 6-day-old larvae. N=5 larvae (487 *mnx1:*GFP^{+}neurons, 590 *vglut2:*DsRed^{+} neurons).
